# Fruit Sizing in Orchard: A Review from Caliper to Machine Vision with Deep Learning

**DOI:** 10.3390/s23083868

**Published:** 2023-04-10

**Authors:** Chiranjivi Neupane, Maisa Pereira, Anand Koirala, Kerry B. Walsh

**Affiliations:** Institute of Future Farming Systems, Central Queensland University, Rockhampton, QLD 4701, Australia; chiranjivi.neupane@cqumail.com (C.N.); maisa.pereira@cqumail.com (M.P.); anand.koirala@cqumail.com (A.K.)

**Keywords:** estimation, fruit sizing, image segmentation, machine vision, measurement, precision horticulture, review

## Abstract

Forward estimates of harvest load require information on fruit size as well as number. The task of sizing fruit and vegetables has been automated in the packhouse, progressing from mechanical methods to machine vision over the last three decades. This shift is now occurring for size assessment of fruit on trees, i.e., in the orchard. This review focuses on: (i) allometric relationships between fruit weight and lineal dimensions; (ii) measurement of fruit lineal dimensions with traditional tools; (iii) measurement of fruit lineal dimensions with machine vision, with attention to the issues of depth measurement and recognition of occluded fruit; (iv) sampling strategies; and (v) forward prediction of fruit size (at harvest). Commercially available capability for in-orchard fruit sizing is summarized, and further developments of in-orchard fruit sizing by machine vision are anticipated.

## 1. Introduction

Forward estimation of orchard fruit load is important for several reasons, including harvest resourcing and market planning. Fruit load estimation requires information on both fruit number and fruit weight. Considerable effort has been placed into the development of methods for the measurement of the number of fruit in orchards in recent years, as reviewed recently by Anderson et al. [1], while the measurement of size of on-tree fruit has been relatively little explored. The last significant review addressing fruit sizing was published over a decade ago [2].

The specifications used in size grading vary by commodity [3]. Weight is a commonly used grading specification, although a specification on lineal dimension is used for the grading of some commodities. The weight of a piece of fruit can be estimated using an allometric relationship between fruit weight and lineal dimensions. Fruit lineal dimensions can be measured non-destructively by several methods, from calipers to machine vision.

Progress in the development of machine vision has been notable in the 14 years since the Moreda, Ortiz-Cañavate, García-Ramos, and Ruiz-Altisent [2] review, both in terms of imaging hardware and the use of deep learning in image analysis. Other enablers include the expansion of broadband communication capabilities, such as 5G into tree crop production areas and the development of cloud computing capabilities. These advances support a suite of fruit sizing applications, ranging from handheld systems based on the ubiquitous mobile devices to specialty mobile imaging rigs which enable the assessment of large areas.

Other considerations relevant to in-orchard assessment of fruit size are sampling and forecasting. While every piece of fruit can be assessed for size on a pack-line, in-orchard assessment involves measurement of a sample of fruit, whatever measurement method is employed. A statistically valid sampling strategy is therefore required. Further, while farm management requires fruit sizing information weeks before harvest, fruit can continue to grow until harvest. Therefore, a growth model is required to allow a forecast of fruit size at the time of harvest from measurements taken some weeks prior to harvest. 

The current review is written to extend the coverage of Moreda, Ortiz-Cañavate, García-Ramos, and Ruiz-Altisent [2], with sections on (i) allometric relations involving fruit weight, (ii) ‘traditional’ methods for measurement of fruit dimensions, (iii) machine vision methods used in measurement of fruit dimensions, (iv) sampling strategies, and (v) fruit growth models for forward estimation of weight at harvest. Case examples are given for mango (*Mangifera indica*) fruit.

## 2. Allometry—Relating Size to Weight

Fruit weight can be based on an estimate of the volume of the fruit, with allowance for the density of the fruit. Where fruit approximate a standard geometric shape, the familiar formulae relating lineal dimensions to volume can be applied, e.g., for a sphere, V=43πr3, where r is radius; for an ellipsoid, V=43π×A×B×C, where A, B, and C are lengths of the three semi-axes; for a cylinder, $V=\pi r^{2}\times L$, where L is length; and, for a rectangular prism, V=L×W×T, where W is width and T is thickness.

Examples of reports relating fruit lineal dimensions or volume to fruit weight using empirically determined relationships are presented in Table 1. Some reports are based on a basic geometric formula relating weight to lineal dimensions, but others are not. For example, Marini et al. [4] implemented a quadratic equation to estimate fruit weight from fruit diameter for three apple cultivars. There is no requirement for an empirically established relationship to follow a physical principle, however greater care must be taken is generalizing the use of the model with fruit from new cultivars or growing conditions.

The empirical relationships established between fruit weight and lineal dimensions incorporate the influence of fruit density, as well as geometric shape. Thus, the use of these relationships in the prediction of new populations of fruit rests on two assumptions: that fruit density and fruit shape are similar in the training and test sets. In practice, fruit shape and density may vary with cultivar, with growth stage and growing condition, e.g., [21,22]. Fruit density can fall below unity when voids form within the fruit and exceed 1 with increased woodiness or starch content. For example, a 10% increase in pineapple fruit density, from 0.94 to 1.03 g/cm^3^, was recorded in the 40 days before harvest, a phenomenon used in the grading by flotation of fruit for certain defects.

Thus, the robustness of a correlation between fruit weight and fruit lineal dimensions must be tested across the range of growing conditions and cultivars for which the relationship is expected to be used. Yuan, Martin, Fullerton, Gould, Hall, and Burdon [9] present a case for modification of the equation coefficient with kiwifruit age, reflecting changes in fruit density and shape. 

The need for such fine-tuning depends on the level of prediction precision and accuracy required by the user. For example, Amaral and Walsh [14] noted an increase in error in using a single model across the mango cultivars ‘Honey Gold’, ‘Calypso’, and ‘Keitt’, as opposed to the use of cultivar-specific models, with RMSE increased from 20.4 to 35.4 g on fruit with an average mass of approximately 550 g. However, the lower RMSE was deemed adequate for the harvest management task.

Loss of measurement precision may also be tolerated in the context of decreased user effort in acquiring fruit sizing data. For example, in the case of mango, most research groups have applied an equation based on the geometrical relationship of volume to the three dimensions of a rectangular prism (Table 1). However, imaging of fruit hanging on tree from one camera perspective can be used to measure fruit length (L), and either width (Wi) or thickness (*T*) if fruit orientation is controlled, or a value between fruit width and thickness if orientation is not controlled. Estimation of fruit weight (W) in these applications requires the use of a simplified relationship, e.g., $W=kLWi^{2}$ for fruit in a controlled orientation, as used in a mobile phone based sizing application [23], with some increase in prediction error, e.g., an increase in RMSE from 23.9 to 25.0 g for mango fruit with average mass of 482 g [14].

The use of machine vision also offers an opportunity to introduce parameters not easily assessed by manual techniques. For example, Utai, Nagle, Hämmerle, Spreer, Mahayothee, and Müller [12] explored use of fruit area in conjunction with L and W for estimation of mango fruit weight. 

In summary, a relationship between weight and lineal dimensions of fruit can often be established, allowing non-destructive estimation of weight of on-tree fruit. Variation in fruit shape and density impact the performance of such relationships, as captured in the RMSE statistic (Table 1). The reported RMSE values are typically around 5% of average fruit mass, with these reports being of training sets. Error of prediction with new fruit populations can be expected to be higher.

## 3. Fruit Sizing with Traditional Methods

**Figure 1:**
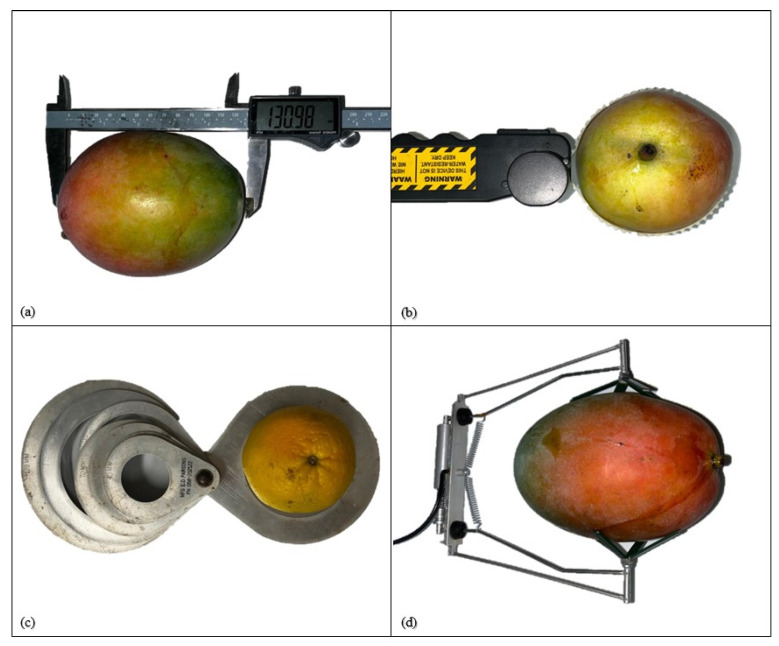
Image of (**a**) digital linear caliper, (**b**) band-loop caliper, (**c**) sizing ring, and (**d**) fruit dendrometer. (Image source: Authors).

The size of fruit on-tree has traditionally been manually estimated using linear calipers, band-loop calipers, sizing rings and dendrometers (Figure 1). These measurements are relative inexpensive, but require operator attention to avoid measurement errors [23] and, typically, are made of a relatively small sample number as the measurements are tedious and time consuming. 

### 3.1. Background

Calipers can be used for the measurement of the distance between two opposite sides of the fruit [24] (Figure 1). Sub-millimeter measurement accuracy is possible, however the placement position of the caliper on the fruit introduces a larger sampling error. Management of this sampling error requires user attentiveness. It is recommended that a measure of variation in repeat measurements of a single fruit be included in reports using calipers, e.g., Wang reported a SD of 1.2 mm on repeated measures of the length of a mango fruit [13].

As the caliper method is labor-intensive, alternative methods are sought [14]; however the method is commonly used to verify the truth of alternate procedures, i.e., as a validation device [14,23,25,26,27,28].

Calipers are available with a visually assessed vernier scale or a digital display. In the last decade, a range of data transfer options have become readily available, avoiding the need for tedious manual data recording. Options include wired and Bluetooth, with the choice of device depending on factors such as convenience, purpose, and cost [29].

### 3.2. Sizing Rings and Band-Loop Calipers

Sizing rings and band-loop calipers (Figure 1) are specialized for measurement of the diameter of spherical fruits, such as oranges, and the girth of cylindrical fruits, such as cucumbers.

Sizing rings are a low-cost option [30] but a very manual measurement option, with a series of rings placed over the fruit to find a size match. Sizing rings are used commercially with a range of fruit and vegetable types, including grapes, bananas, peaches, and carrots [30]. Calibration ring openings are usually circular, but a square shape is used in measurement of potatoes and onions [31]. Smaller fruits, such as grapes and cherries, also have ‘sizer card’ style options commercially available [32].

The United States Department of Agriculture [33] provides specifications on openings and gauge thickness of sizing rings. For example, plastic rings can have a maximum opening of 33 mm and a minimum thickness of 1.4 mm. Aluminum rings do not have a minimum diameter, but they must have a minimum thickness of 1.5 mm.

Band-loop calipers are typically used to assess the largest circumference of the fruit, with fruit diameter inferred from the measured circumference [6]. Band-loop calipers offer some advantage over standard calipers regarding placement variation issues, as they measure perimeter, not a point position.

### 3.3. Dendrometers

Dendrometers are a special case of caliper involving continuous monitoring, A typical design consists of a linear voltage displacement sensor within a spring-loaded assembly placed over a fruit (Figure 1). The growth of fruit can be continuous over months without affecting the size of individual fruit growth [24]. For example, dendrometers were used by Morandi et al. [34] to observe the influence of water stress on the change in fruit size of on-vine kiwifruit and by Fernandes et al. [35] to observe change in fruit size of olive fruit. Phytek (Kfar Saba, Israel) produces LoRa-enabled fruit dendrometers for use within an irrigation scheduling management system.

The accuracy of dendrometers is directly related to the quality and, thus, the cost of the instrumentation. Maintenance is required, particularly to correct movement of the sensor position on the fruit following disturbances, e.g., wind [24]. Due to their cost and maintenance requirements, dendrometers are typically used in low replicate numbers. The technology is thus unsuited to assessment of a size profile of a fruit population but well suited for assessment of the rate of fruit growth, although relevance to the orchard fruit population depends on the choice of fruit representative of the orchard.

## 4. Fruit Sizing with Machine Vision

### 4.1. Background

RGB imagery is adequate for fruit identification (detection or segmentation) and on-tree fruit count, e.g., in mango orchards, [36] and [1]. For fruit sizing, however, the dimension of a detected object must be converted from image pixels to real world dimension. A ‘first generation’ approach involved placement of an object or scale with known dimensions in the field of view [23,27,37], or acquired images at a known camera to object distance [38,39]. A ‘second generation’ approach involved the use of the combination of RGB and depth sensors in a single (RGB-D) camera to obtain camera-to-object distance information. Both approaches to fruit segmentation are undertaken using the 2D RGB image. In a ‘third generation’ approach, segmentation can be based on 3D point clouds generated from RGB-D or LiDAR data. The 2D method involves the imaging of a given fruit from a single camera perspective, with conversions of a measurement of the lineal dimensions of the height and width of the imaged fruit in pixels to metric dimensions. The 3D method involves the imaging of a given fruit from several perspectives to allow the generation of a reconstruction. Size metrics are then assessed of the reconstruction. Other advances have occurred in the methods used in fruit detection and segmentation.

Fruit may be partly occluded by leaves or other fruit in images of whole tree canopies. Detected partly occluded fruit should be included in fruit counting applications, but for a fruit sizing pipeline these detections should either be rejected, e.g., as undertaken by [13] and Neupane et al. [40], or the geometry of the fruit must be reconstructed from visible portions of the fruit, e.g., as undertaken by Wang and Chen [41], Gené-Mola et al. [42], and Mirbod, Choi, Heinemann, Marini, and He [28].

Table 2 provides a summary of the studies identified in a search of the Scopus database using the search parameters “TITLE (fruit AND siz* AND (measurement OR estimation) AND NOT cell) OR KEY (fruit AND siz* AND (measurement OR estimation) AND NOT cell) AND PUBYEAR > 2008 AND PUBYEAR > 2008 AND (LIMIT-TO (SUBJAREA, “AGRI”) OR LIMIT-TO (SUBJAREA, “ENGI”) OR LIMIT-TO (SUBJAREA, “COMP”))” for the period 2009–2023. Further searches were conducted in ‘Google Scholar’ using various combinations of keywords ‘fruit’, ‘vegetable’, ‘size’, ‘sizing’, ‘estimation’, ‘prediction’, ‘measurement’, ‘3D’, ‘point cloud’, and ‘depth camera’. Out of a total of 42 papers published between 2009 and 2023 that discussed use of machine vision-based sizing, a total of 17 studies used the 3D method for sizing, with 14 studies mentioning the used of RGB-D depth cameras. In terms of application context, a total of 22 studies were conducted in a laboratory environment, 4 in a green house, 4 in a packhouse. Two studies implemented a stand-alone application on a mobile device with use of an external scale.

### 4.2. Application Scenarios

Fruit size estimation using machine vision has been implemented in fruit pack-lines since the 1980s, e.g., [68]. Commercial vision systems in pack-line applications utilize a structured imaging environment, e.g., fixed camera angles and distances and use of a lighting box with optimum illumination, to facilitate vision assessment of fruit attributes. Multiple cameras are typically employed, providing multiple perspectives of each fruit, and roller cups or conveyors can rotate the fruit as it passes under the field of view of the camera. 

In contrast, imaging conditions are far less controlled in an orchard setting. Image quality in daytime is significantly affected by a range of illumination conditions across the image, from over-exposure to strong shadow. This issue is exacerbated in the strong sunlight of tropical settings. Artificial lighting can be used to provide consistent imaging conditions, either as high-intensity strobe lighting with very short exposure time to reduce the effect of sunlight [69] or by image acquisition at night time images with lower cost lighting [1].

**Figure 2:**
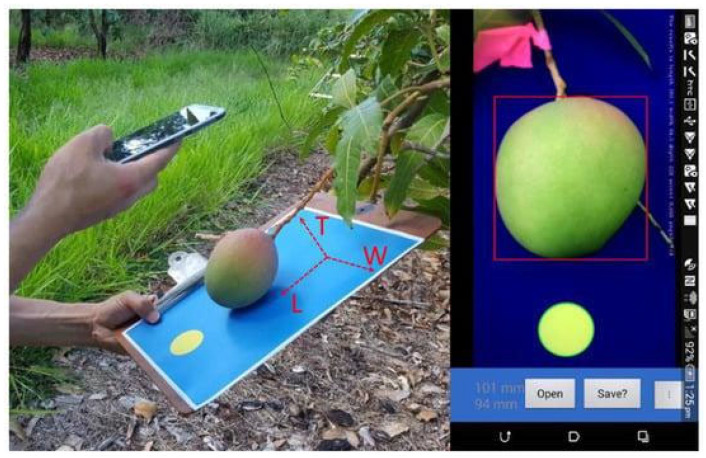
Use of a mobile phone in size estimation of fruit on tree images. The yellow circular marker is used as a scale for fruit sizing and the blue background provides better contrast for fruit segmentation in images. (Image source: [23]).

**Figure 3:**
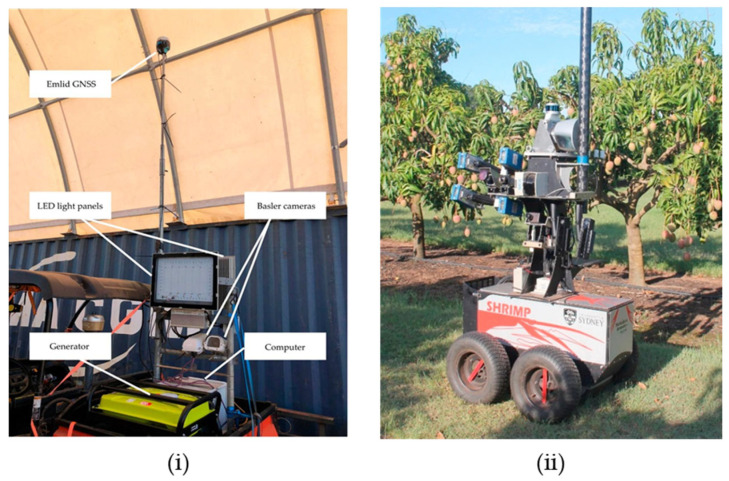
Imaging system mounted to a vehicle for on-the-go image acquisition. (Image source: (**i**) [1]; (**ii**) with permission [70]).

For in-orchard fruit size estimation, three application scenarios have been reported: (i) the use of a smartphone or tablet as a handheld imaging device (e.g., Figure 2) with sufficient computing power for image processing or communication capacity to enable cloud processing; (ii) the use of a depth camera mounted to a mobile platform (e.g., Figure 3) which moves through orchard inter-rows; and (iii) a camera in a fixed position, used for continuous measurement of fruit size.

Publications on the use of handheld imaging solutions have reported use of a physical marker as a scale for inference of fruit dimensions (Figure 2). For example, Wang, Koirala, Walsh, Anderson, and Verma [23] employed a backing board that incorporated a scale, placed behind the hanging fruit. The fruit was positioned relative to the camera such that fruit length and width were captured in the image. An RMSE of about 4 mm on measurements of both fruit length and width was achieved. Application issues include strong lighting, and tilt and yaw of the camera relative to the object plane. Possible improvements include use of a fixed frame to hold the camera parallel to the backing plane, although this reduces the portability of the system. 

From the published work (that involves inclusion of a scale in the image), there appears to be little advantage in sampling speed or ease of use of the mobile device fruit sizing application compared to use of calipers with a data transfer/storage option, although there is advantage in technology accessibility—mobile devices are ubiquitous. 

In the second application, images are collected in a ‘drive-by’ mode, as performed for fruit number estimation, e.g., [13,40,63]. RMSEs of 4.9 and 4.3 mm on fruit length and width, respectively, were reported for the system employed by Wang, Walsh, and Verma [13]. The advantage of this system over the handheld system lies in the ability to upscale to an orchard level given the ability to rapidly collect image data. Disadvantages include (i) a higher capital cost; (ii) a potential for higher RMSE arising from the increased camera to fruit distance and uncontrolled fruit orientation; and (iii) a potential sampling bias if a size difference exists between the fully visible fruits processed for sizing and remaining partly and fully occluded fruit.

In a third application, real-time monitoring of fruit growth using machine vision estimation of fruit size was reported by Behera, Sethy, Sahoo, Panigrahi, and Rajpoot [47]. This system employed a fixed position camera with a 4G internet connection to a remote server for processing.

### 4.3. Hardware for Machine Vision-Based Fruit Sizing

Fruit can be localized and segmented within an RGB image, with object length and width measurement made in units of image pixels. The conversion of object pixel dimensions to real world dimensions can be undertaken using the input of the camera to fruit distance, which can be assessed using LiDAR, one of the expanding ranges of RGB-D cameras, or other depth sensing technology. 

A number of studies have compared the performance of depth camera technologies (stereovision, Time of Flight (ToF), structured light, and active IR stereo) in the context of application scenario [13,71,72,73,74,75,76]. For example, ToF cameras provide better depth accuracy than stereovision [13,74], but the technique is not recommended for use in strong sunlight. Other factors, such as the field of view (FoV) of the depth sensor, frame capture rate, use of color or monochrome imagery, and weather proofing also impact choice of hardware for the use case of in-orchard measurement. Commercial product life is also a consideration, as exemplified by the Microsoft Kinect v1 and v2, which each entered and exited the market within a period of 5 years.

Combination RGB—Time of Flight (ToF) depth cameras have dominated horticultural sizing applications. For example, the Kinect v2 was used in the sizing of mangoes [11,13], onions [65], citrus fruits [51], and pears [41]; the ToF RealSense L515 was used for sizing of peppers [57] and apples [46]; and the PMD CamCube 3.0 ToF camera was used by [25] for apple fruit size estimation (Table 2). The successor in the Kinect camera series, the Azure Kinect ToF camera was released in 2019 with improved depth and RGB sensor resolutions, good angular resolution, lower noise, and better accuracy [77]. The Azure Kinect camera was used for mango fruit sizing by Neupane, Koirala, and Walsh [40]. 

Depth cameras based on active IR stereoscopy technology have also been used for fruit sizing, e.g., the Intel RealSense D435 for use in grape cluster sizing [63], peach fruit sizing [78], RealSense D415 for cucumber, eggplant, tomato, and pepper sizing [55] (Table 2). The ZED mini stereo camera was used for sizing of tomato fruit by Hsieh, Huang, Hsiao, Tuan, Shih, Hsieh, Chen, and Yang [54].

Neupane, Koirala, Wang and Walsh [74] evaluated accuracy of eight depth cameras of various technologies for the application of in-orchard fruit localization and sizing. The Azure Kinect was recommended in terms of depth accuracy, outdoor use, cost, and its integrated RGB-D capability. The Blaze 101 (Basler, Ahrensburg, Germany) was recommended for its relative insensitivity to daylight, by use of 940 rather than 850 nm illuminating light, and its IP67 rating.

### 4.4. Software for Machine Vision-Based Fruit Sizing

Fruit size estimation using machine vision requires object detection, followed by extraction of pixels belonging to fruit using a color- or intensity-based threshold or deep learning-based segmentation method. Having segmented the object of interest, either the ‘2D’ or ‘3D’ method, can be applied. These topics are covered in this section.

#### 4.4.1. Image Segmentation

**Figure 4:**
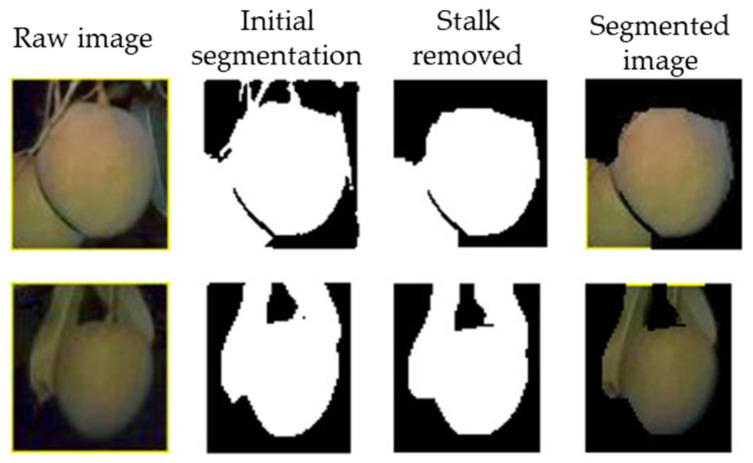
An example of mango fruit segmentation using Otsu’s thresholding. In image 1 (top row), correct segmentation of the fruit is achieved. In image 2 (bottom row), leaves have been segmented with the fruit (Image source: [40]).

One of the common approaches used for segmentation of fruit pixels in an image is thresholding. Thresholding involves setting a threshold value for pixel intensity, with categorization of pixels above or below the threshold into fruit or background pixels. For example, a segmentation method based on a threshold set from a grayscale histogram, Otsu method [79] has been used for fruit segmentation by Wang and Li [65] for sweet onion; Wang, Walsh, and Verma [13] and Wang, Koirala, Walsh, Anderson, and Verma [23] for mangoes; Gongal, Karkee, and Amatya [25] and Lu, Chen, Zhang, and Karkee [27] for apples; and by Ponce, Aquino, Millán, and Andújar [59] for olives. Thresholding methods, including Otsu segmentation, fails if the object of interest (fruit) and background objects have similar characteristics, e.g., color and texture (Figure 4), with a false segmentation mask resulting in a false sizing result. Other color- and intensity-based thresholding methods, e.g., [64], can also fail to properly segment fruit pixels from the background. 

**Figure 5:**
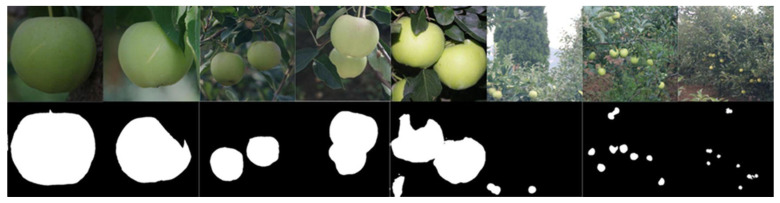
An example of U-Net based semantic segmentation of green apple on tree at several image scales. (Image source: with permission [81]).

An alternative approach involves use of a CNN based semantic segmentation network, such as U-Net [80]. Semantic segmentation algorithms categorize image pixels into classes but do not separate instances of the same class. This limits the use of the technique in sizing of fruits that overlap in bunches/clusters (Figure 5). For example, Fukuda, Okuno, and Yuki [56] used a U-Net based segmentation method for segmentation and sizing of on-tree pear fruit (Figure 5). 

**Figure 6:**
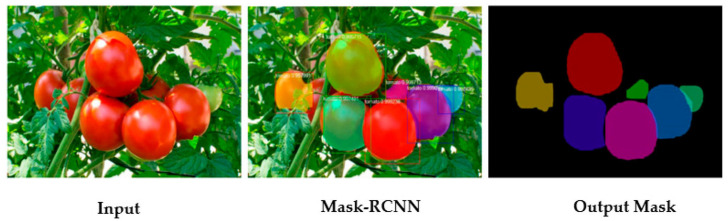
Example of Mask R-CNN segmentation, with 7 of 8 fruit segmented as represented by colored masks in right side image (Image source: [53]).

CNN instance segmentation networks are capable of segmenting pixels belonging to different object classes and separating instances of each object class. Mask R-CNN [82] is a popular instance segmentation network based on the two-stage detection method of R-CNN [83]. Mask R-CNN was used for segmentation and sizing of tomato by Lee, Nazki, Baek, Hong, and Lee [53] (Figure 6) and Hsieh, Huang, Hsiao, Tuan, Shih, Hsieh, Chen, and Yang [54]; for mango by Neupane, Koirala, and Walsh [40]; and for apple by Mirbod, Choi, Heinemann, Marini, and He [28]. YOLOv8 (https://github.com/ultralytics/ultralytics, accessed on 8 March 2023) is a recently developed object detection and instance segmentation network which offers better speed based on the one-stage detection method of YOLO. This network is recommended for applications requiring real time fruit sizing.

**Figure 7:**
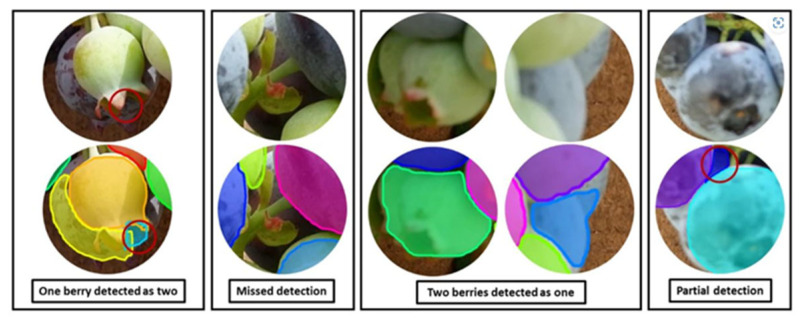
Types of instance segmentation error indicated by red circles and colored segmentation masks (Image source: [84]).

Following the separation of object (fruit) instances using instance segmentation technique, a check is required to verify whether each instance is a shape mask of a complete fruit or a mask of a partly occluded fruit. Figure 7 illustrates an instance segmentation failure in the context of fruit occlusion. Sometimes, it is possible that instance segmentation network to generate false segmentation masks. Ni et al. [84] provided examples of four different cases of segmentation failure, such as multiple fruits segmented as one, missed fruits, single fruit detected as two, and partial segmentation of a fruit (Figure 7).

A list of papers addressing fruit sizing using machine vision is categorized by the segmentation method used in Table 3.

#### 4.4.2. 2D Segmentation

The 2D method involves measurement of the lineal dimensions of the detected object in terms of image pixels, followed by conversion of pixel to real dimensions. This conversion can be based on the use of a reference scale placed in the image plane of the object, or by use of a camera pin-hole model, given camera to fruit distance and use of the thin lens formula and intrinsic camera parameters, such as focal length. As noted earlier, camera to fruit distance can be obtained using RGG-D cameras using either stereo-vision, structured light, ToF, or active infrared (IR) technologies. 

Example applications follow, chosen to represent a progression in the evolution techniques in the 2D method.

A ‘first generation’ approach to scaling involves placement of an object of known size in the object plane, as illustrated by the work of Wang, Koirala, Walsh, Anderson, and Verma [23] involving an app on a mobile device, with imaging of fruit against a backing board of a contrasting color which positioned the fruit relative to the camera, such that fruit length and width were captured in the image. A circular marker on the board was used as a scale, with a correction for the difference in the plane of the fruit perimeter and the scale based on an allometric relationship between fruit length and thickness. For sizing of citrus fruit in canopy images, Apolo-Apolo, Martínez-Guanter, Egea, Raja, and Pérez-Ruiz [37] placed a rectangular marker of known size into the canopy, to act as a reference scale. Kohno, Ting, Kondo, Iida, Kurita, Yamakawa, Shiigi, and Ogawa [49] developed a mobile citrus fruit size grading system in which the camera to fruit distance was fixed. 

In a variant on the inclusion of a scale in image, Hsieh, Huang, Hsiao, Tuan, Shih, Hsieh, Chen, and Yang [54] used the ratio of bounding box dimensions of detected tomato fruit in images to the physical dimensions of sampled fruit as a calibration factor to predict fruit sizes on new images.

A ‘second generation’ approach to image scaling involves use of a RGB-D camera to obtain camera to object distance information, as illustrated by the work of Wang, Walsh, and Verma [13] using depth information from the Kinect v2 RGB-D camera for conversion of bounding box pixel dimensions into real world dimensions. Similarly, Kurtser, Ringdahl, Rotstein, Berenstein, and Edan [63] employed depth information from the RealSense D435 RGB-D camera to estimate grape cluster size and Bortolotti, Mengoli, Piani, Grappadelli, and Manfrini [78] used distance measurements from the RealSense D435 and D455. 

In a variant on this approach, Wittstruck, Kühling, Trautz, Kohlbrecher, and Jarmer [67] presented estimated size of pumpkin fruit from high-resolution aerial imagery of a UAV flown at a known height above the ground. Measurement error in this application is relatively high, with a reported standard deviation on measurement residuals of 3.0 cm on fruit diameter, for fruit with a mean diameters of 13.8 cm. This error could be associated with low pixel resolution and/or error in height measurement.

Other advances have occurred in the methods used in fruit detection and segmentation, as described in the previous section. A ‘first generation’ approach is illustrated by Kohno, Ting, Kondo, Iida, Kurita, Yamakawa, Shiigi, and Ogawa [49] who created binary masks for citrus fruit generated using a simple color thresholding technique. Similarly, Wang, Koirala, Walsh, Anderson, and Verma [23] used a binary mask of mango fruit obtained using Otsu’s dynamic thresholding, then a morphological operator to remove the fruit stalk before extracting pixel dimensions. Patel, Kar, and Khan [48] used HIS color space thresholding to obtain contours of mango fruits.

A ‘second generation’ approach to fruit detection and segmentation involves the use of machine learning for object detection, with fruit dimensions taken from a fitted bounding box. For example, Wang, Walsh and Verma [13] used a cascade classifier model trained on histogram of oriented gradient (HOG) features, followed by Otsu’s thresholding. Apolo-Apolo, Martínez-Guanter, Egea, Raja, and Pérez-Ruiz [37] used the deep learning Faster R-CNN model for fruit detection on images of tree canopies. 

Kurtser, Ringdahl, Rotstein, Berenstein, and Edan [63] reported that of four algorithms trialed, viz., percentile bounding box edges, percentile bounding box diagonals, ellipsoid fitting, and cylinder fittings, the lowest average absolute error was obtained using enclosing bounding box on refined segmentation through color-based K-Means clustering.

A ’third generation’ approach involves deep learning-based semantic or instance segmentation to obtain object perimeters. For example, both [53] and [54] used the Mask R-CNN instance segmentation method to extract tomato fruit masks from images. Fukuda, Okuno and Yuki [56] utilized a deep learning semantic segmentation method (UNet). Zaenker, Smitt, McCool, and Bennewitz [57] employed the instance segmentation method (YOLACT) to extract masks of pepper fruit.

There is a general trend for improvement in reported measurement accuracy and precision with these ‘generations’ of technology, although direct comparison of published results is compromised by the use of different image sets and the reporting of different performance metrics (Table 2). An RMSE of < 5 mm is now routinely achieved on measurement of lineal dimensions of fruit size for non-occluded fruit. For a ‘best case’ result involving use of controlled-lighting against artificial plain background and a high camera resolution [53], mean average errors of 2.3 and 2.6 mm for fruit length and width estimates, respectively. This result likely represents the error of the reference method, caliper measurements, with a SD of repeated measurements of around 2 mm reported by Anderson et al. [86].

It is recommended that the performance metric of RMSE always be reported. To facilitate inter-study comparisons. The public release of an RGB-D data sets for a number of commodities and imaging conditions is also recommended, to allow direct comparison of new techniques. 

#### 4.4.3. 3D Segmentation

Fruit segmentation on 2D image data fails when fruit are visually similar data (in shape, color, and texture) to the background, although instance segmentation can improve results markedly. The 3D information can also assist in segmentation. The 3D point clouds can be generated from RGB-D data, with a method required for identifying the cluster of points associated with the object of interest, i.e., fruit. Information from multiple image captures, involving multiple perspectives of the fruit, can also be combined in generating the 3D point cloud. 

Color can be used as a criterion in 3D segmentation. Lin, Tang, Zou, Xiong, and Fang [51] reported color based segmentation of the 3D point cloud from RGB-D images of citrus fruit on tree using a Bayes-classifier, followed by grouping of adjacent points using a SVM classifier and a density clustering method. Wang and Chen [41] clustered RBG-D point clouds associated with pear fruit within canopy images, using the locally connected convex patches (LCCP) method, followed by use of a principal component analysis bounding box algorithm to acquire morphological features of the fruit. 

Gené-Mola, Sanz-Cortiella, Rosell-Polo, Escolà, and Gregorio [42] used structure from motion (SfM) and multi-view stereo (MVS) methods for generation of a 3D point cloud in an on-tree apple fruit sizing application. Of the methods of M-estimator sample consensus (MSAC), template matching and least squares, the template matching technique provided the lowest mean absolute error (MAE) for occluded fruits. In a banana fruit study, Hartley, Jackson, Pound, and French [38] used a 3D reconstruction method based on cycleGAN (generative adversarial network-based model). Zheng, Sun, Meng, and Nan [55] used a key-point RCNN model to identify six key points on vegetables from input color images, with mapping of the key points to a 3D coordinate system to obtain physical dimensions. Freeman and Kantor [46] used a YOLACT instance segmentation model for fruit detection, segmentation, and ROI generation. The 3D fruit surface from the point cloud was generated using the DBSCAN clustering algorithm and the axes of a fitted ellipse were used as dimension measures for fruit sizing. A comparison was made between the ‘ROI Viewpoint Planner’ (RVP) and the ‘Fruitlet Viewpoint Planner’ (FVP) methods, with the latter recommended for lower error in the sizing result. Future work should see consensus emerge on a recommended technique.

The 3D segmentation method has an increased computation requirement compared to the 2D methods, which can be problematic. For example, an average time of 1.25 s was reported for identification and localization of an individual fruit in the computing hardware used by Lin, Tang, Zou, Xiong, and Fang [51]. 

Using ‘ideal’ conditions (of optimal indoor lighting and imaging of fruit of a turntable to capture multiple perspectives), Wang and Chen [41] reported a RMSE of 1.17 and 1.03 mm on pear fruit diameter for fruit height and diameter for sizing based on segmentation of the 3D point cloud. *X*, *Y*, and *Z*-axis positional errors of 7, −4, and 13 mm, respectively, was reported for fruit localization using the 3D point cloud of citrus fruit on trees [51], with a bias of −1 mm and a median absolute deviation of error of 4 mm reported in fruit size estimation. Using RGB-D images collected at an approximate camera to fruit distance of 1 m, MAPE on length measurements was 14.2% for cucumber, 7.4% for eggplant, 11.6% for tomato, and 14.5% for pepper. At an approximate fruit length of 100 m, these values are equivalent to errors of around 10 mm. Freeman and Kantor [46] reported a MAE of 1.04 mm in their application.

In summary, a major attraction to the use of the 3D method is its potential to improve segmentation of occluded fruit and to improve sizing accuracy, as indicated in the work of Gené-Mola, Sanz-Cortiella, Rosell-Polo, Escolà, and Gregorio [42].

### 4.5. Dealing with Occlusion 

As noted earlier, occluded fruit in images of fruit on-tree must be excluded from size analysis, or a morphological operator employed to reconstruct the outline of the entire fruit. 

**Figure 8:**
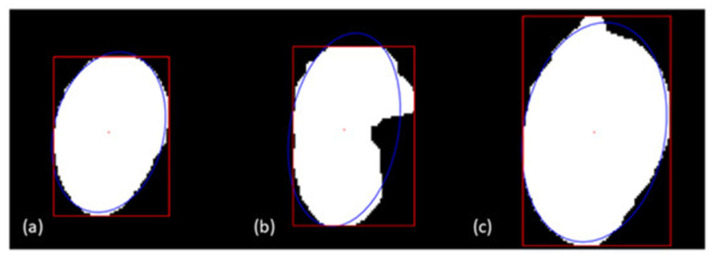
Occlusion handling with ellipse fitting. (**a**) represents well separated fruit, (**b**) and (**c**) represent unsuccessful criteria for ellipse fit. (Image source: [13]).

Partly occluded fruit within 2D images have been excluded from analysis following identification using various geometric rules. For example, Wang, Walsh, and Verma [13] and Neupane, Koirala, and Walsh [40] used ellipse fitting on segmentation masks and used a pixel area overlap threshold to validate fully visible (complete) fruit (Figure 8). Occluded fruits were also filtered on the basis of pixel mask area, depth values of the objects and the ellipse eccentricity value.

In another approach using 2D image data, Mirbod, Choi, Heinemann, Marini, and He [28] attempted to train a neural network to classify fruit as non-occluded and occluded fruits. However, false classification rates were relatively high, impacting size estimation.

**Figure 9:**
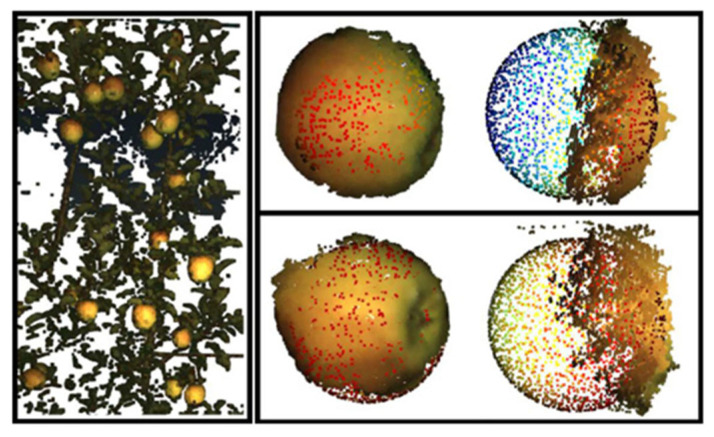
Sphere fitting on point cloud. Top and bottom right images represent 3D reconstruction from point clouds with sphere fitting. (Image source: with permission [28]).

Template matching or geometry reconstruction approaches have been used with circular symmetrical fruit, such as blueberries, citrus fruit, and apples for both 2D and 3D shape fitting, e.g., [41,42]. Gené-Mola, Sanz-Cortiella, Rosell-Polo, Escolà, and Gregorio [42] report an MAE of 3.7 mm on apple fruit diameter using the M-estimator sample consensus (MSAC) algorithm-based sphere fitting method. Similarly, Mirbod, Choi, Heinemann, Marini, and He [28] used a sphere fitting approach using point clouds for apple fruit diameter estimation, reporting an MAE of 3.93 mm. The authors indicate that noisy point cloud data along the fruit surface expanded the fitted sphere, contributing to the error in fruit diameter estimation (Figure 9).

### 4.6. Commercial Offers for Fruit Sizing

As the technology for machine vision-based fruit sizing becomes more mature, commercial tools begin to be offered for grower use (Table 4). These systems involve a diverse use of technologies—from use of the RGB and depth cameras in consumer grade handheld mobile devices to specialty imaging systems mounted on mobile ground or aerial platforms. Some systems use edge computing capacity for in-field image processing, while others rely on transfer of data for cloud processing. Some systems employ 2D processing techniques, while others have employed 3D processing. The range of technologies offered is highlighted in the following examples.

CropTracker^TM^ offers a tablet-based system. Images of fruit in harvest bin are acquired at a distance of around 50 cm scan, with 3D reconstruction occurring in the cloud. Fruit diameter estimates within 1–3 mm of actual are claimed.

Spectre^TM^ is deployed on a smart tablet or phone or as a camera on a fixed portal frame, imaging the top layer of fruit in trucks. An image of harvested apple fruit in a field bin is processed using a deep learning model for fruit detection, and sizing is based on the known dimensions of the field bin. A perspective transformation is used to adjust the image of the field bin into a ‘top–down’ view, to accommodate operator difficulty in holding the camera parallel to the object. A color calibration feature allows adjustment for the lighting environment (outdoors vs. indoors). The app outputs a fruit count and a size distribution for the top layer of fruit. 

Harvest Quality Vision^TM^ is deployed either as an RGB-D enabled tablet equipped with a macro lens for imaging of fruit in field bins, or as an array of cameras on a fixed portal frame, imaging the top layer of fruit in trucks. The handheld application involves the acquisition of multiple images of fruit in field bins over approximately 3 s at a camera-to-fruit distance of 50 cm. A 3D reconstruction is undertaken on a cloud-based processor, providing color, quantity, and size information. A 1–3 mm sizing accuracy is claimed, enabled by the close camera-to-fruit distance and the 3D reconstruction. 

Pixofarm^TM^ offers fruit count and sizing capability deployed on a mobile device. A sticker must be affixed to each fruit to be measured, providing a scale (akin to the approach of Wang, Koirala, Walsh, Anderson, and Verma [23]). An output of average size, size class distribution and growth rate are provided.

FruitScout^TM^ is an app delivered through a mobile device for estimation of trunk diameter, and bud, blossom, and fruit count and size [87].

Aerobotics^TM^ offers an app for an iPhone device with three cameras for monitoring yield and size estimation (Fresh Plaza, 2022). Images are uploaded to a cloud server for processing, with a claimed error of within 1.5 mm. A UAV based solution is used for orchard imaging, providing ‘Smart Sampling Locations’. for fruit size measurements, with the app used to guide scouts to geo-referenced locations.

Tevel Aerobotics^TM^ is developing a tethered UAV based apple harvester. The UAV is equipped with an RGB-D camera (RealSense D415) that is used in estimation of fruit size at the time of harvest.

The commercially available systems are evolving rapidly with improvements in consumer sensors in mobile devices and in cloud computing and communication network coverage of orchards. For example, it is notable that a number of the commercial products use cloud processing of images. Additionally notable is the quick adoption of the LiDAR camera introduced with the iPhone 12 and iPad Pro [88] into fruit sizing apps.

A commercially relevant solution can differ from a researcher’s solution for farm implementation reasons. For example, mobile phone processor capacity now allows for image processing using lightweight deep learning models, e.g., [89], but a practical implementation involving large numbers of images can be problematic. Thus, a number of commercial solutions involve cloud processing. 

The merging of different solutions in the commercial products is also encouraging, e.g., in-field fruit sizing with a system for representative sample selection and location, and fruit sizing at harvest. However, continued research is required to validate the solutions being proposed.

## 5. Sampling 

### 5.1. Sampling Strategies

Not every fruit in the orchard can be measured, as some are on a pack line after harvest, so in-orchard measurement is undertaken of a sample of fruit. The need to sample is an obvious requirement when using methods that involve measurement of a small number of fruit per orchard, whether using a traditional method, e.g., calipers, or a technique such as handheld machine vision. However, the need is also true for machine vision implemented on a mobile platform, as operator effort can be reduced by driving a sample of inter-rows in the orchard. 

There is spatial variability in fruit size, both between fruit on a tree and between trees [90,91]. For example, a delay in flowering on one side of a canopy or area of the orchard will result in smaller fruit in those locations, or fruit inside the tree canopy may be delayed in maturation compared to fruit in the outer canopy. As this variation is not spatially uniform, a strategy is required for the acquisition of a sample that represents the orchard population of fruit. As sampling error can often exceed measurement error, effort spent characterizing the source of fruit size variation within an orchard (between and within trees) will be well spent in informing the design of a sampling strategy to deal with this variance.

The pioneering study by Pearce [92] with apples and pear crops provides a review of the sampling techniques of random sampling, stratified sampling, systematic sampling, and cluster sampling, with considerations of required sample size, sampling frequency, and measurement precision. Recent reviews of the topic can be found by Anderson, Walsh, and Wulfsohn [1] and Walsh et al. [93] The most common sampling strategies currently applied in the assessment of orchard attributes are:

1. **Random Sampling:**
  (i) With replacement: In this approach each “member” of a population has an equal and independent chance of being selected for the sample. It is an inefficient sampling design, but it provides independent samples, simplifying statistical analysis; and
  (ii) Without replacement: When the population is large, the chance of selecting the same unit more than once is low, and the results are effectively identical to those obtained from sampling with replacement.
2. **Systematic Sampling** is a sampling method using a systematic grid, e.g., sampling from every 13th tree. The regularity of systematic sampling is much more convenient for the operator than random assignments in terms of locating allocated sample positions.
3. **Stratified sampling** involves the division of the population into externally heterogenous but internally homogeneous groups or ‘strata’, which are then considered separate populations. This method offers an advantage if the criteria for stratification can be easily applied, and if the variance of the attribute of interest is decreased within the strata, leading to an overall lower sampling requirement. Different sample numbers are used for each stratum, reflecting the attribute variance within those strata.
4. **Cluster sampling** involves the division of the population into externally homogeneous but internally heterogeneous ‘clusters’. Each cluster contains variation similar to that within the entire population.
5. **Multi-stage sampling** involves the division of the population at two or more ‘levels’, with application of one of the probability sampling methods mentioned above at each level. Different sampling methods can be applied at each stage. For example, orchard blocks may be sampled of a farm, trees may be sampled of selected blocks, and fruit may be sampled of selected trees.

In horticultural practice, management of a given fruit-tree farm is divided into orchard units, typically of around 1000 trees, of relatively uniform tree condition and management practices. For sampling purposes, orchard units of very similar tree condition and management may be grouped together. This grouping represents the stratification of the farm. A single unit from a given management group could be chosen for sampling, with measurements to represent the whole group—a cluster sampling approach. To obtain an estimate of fruit size population mean and variance within each unit, a sample of fruit must be chosen using a probability sampling method. A direct application of the random sampling method would involve the allocation of numbers for every item of fruit in the orchard, which is obviously impractical. Rather, a sample of trees may be first selected, and then a sample of fruit from the selected trees can be measured for size, with a probabilistic sampling method used at each stage.

Each orchard unit may be further divided into strata or clusters. Stratification is more applicable than clustering for this application. Stratification requires a correlation between the attribute of interest, fruit size, and an attribute for which data are easily collected. Trees with each stratum must then be chosen using a probabilistic method. Tree canopy area or Normalized Difference Vegetation Index (NDVI), from satellite or unmanned aerial vehicle (UAV) imaging, have been used in such stratification exercises. However, NDVI stratification, followed by random sampling, did not reduce the sampling effort (number of trees) in the estimation of fruit load in a mango orchard [86]. In contrast, NDVI images were used to select representative sampling locations, using a modified heuristic algorithm to determine the most efficient protocol [94]. NDVI stratification followed by random sampling was more efficient than simple random sampling for the two vineyards considered, with sample size requirement reduced by up to 69% and distance travelled between sampling locations reduced by 93%.

**Figure 10:**
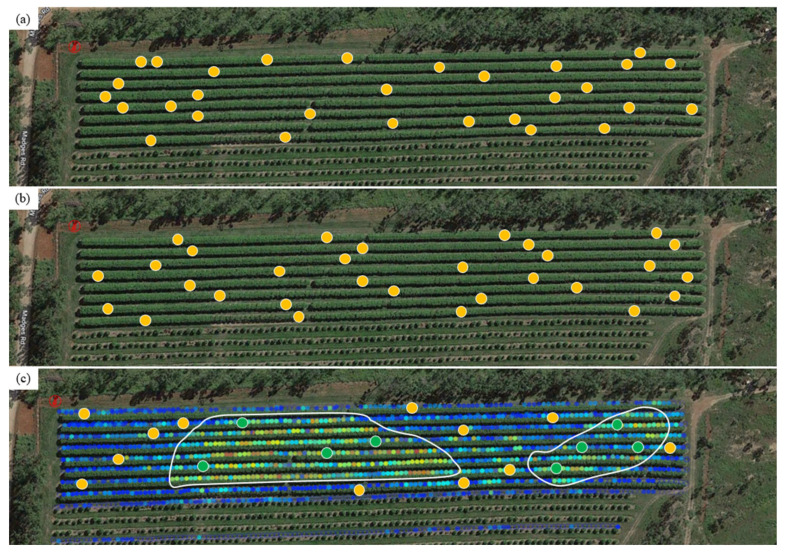
Sampling of trees with an orchard of 968 trees using (**a**) random sampling (*n* = 30), (**b**) stratified sampling (*n* = 30), based on selection of every 32^nd^ tree from a random starting tree within the first 32 trees of the first sampled row, and (**c**) random sampling within two areas (*n* = 12 (yellow dots) and 8 (green dots)) following stratification based on a machine vision estimation of early fruit set, capturing fruit of a first flowering event. Warmer colors, from blue through yellow to red, represent higher fruit count per 4 m of row.

Other criteria may also be applied in stratification. An obvious criterion that is associated with fruit size is time of flowering. For example, different areas of the orchard may vary in flowering time (Figure 10, bottom panel), allowing stratification at a tree level. In the Figure 10 example, reduced variance in fruit size within the two stratified areas (compared to variance across the whole orchard) allowed reduction in sample size. Alternatively, more than one flowering event may occur across all trees. In this case, stratification can be applied at a flowering event level if the size of fruit from the two events is sufficiently different as to allow unambiguous classification (typically associated with separate harvest events).

Random sampling techniques can be difficult to implement routinely in terms of the effort required by an operator to locate the random positions. This difficulty can be addressed at the level of tree selection within the orchard by use of geolocation technology within mobile device apps, to guide operators to the selected positions. Alternatively. sampling of every x^th^ tree in an orchard (systematic sampling) is more efficient for an operator, being easier for location of target trees (Figure 10) [95].

However, while the random assignment of row and tree number is straightforward, a truly random selection of fruit on a tree is problematic. For example, to affect a random sampling of fruit on an apple branch, De Silva et al. [96] numbered all fruit and selected based on a random number draw. A systematic sampling (as every k^th^ fruit in order within the branch) method was recommended as being more efficient than random or stratified (on position along length of branch) sampling of apple fruit on a limb for estimation of mean fruit weight, but random sampling was recommended for estimation of population variance.

Martínez Vega et al. [97] has advocated the use of a multi-stage sampling technique termed Systematic Uniform Random Sampling. This method involves systematic sampling of trees, branches and branch segments, and simple random sampling of fruit within the branch segment [97]. The periodicity of sampling requires prior knowledge of the source of variation (within vs. between trees). The method involves measurement of a high number of the sampling units, distributed through the volume of the tree canopies. The method resulted in a decrease in sample size compared to a random sampling strategy [1] and made the task of locating sampling units (tree branch segments) easier. However, implementation requires a reasonably well-structured tree canopy, to enable identification of branch hierarchy.

In horticultural practice, a pseudo-random strategy is often applied, although it is recognized that such a selection of fruit from an entire tree canopy is likely to include an operator bias, with an unconscious selection of smaller or larger fruit, or fruit from an (accessible) area of the canopy that are not representative of the whole canopy. For example, Marini [98] reported a selection of 20 ‘representative’ fruit from the periphery of each tree at a height between 1.5 to 2.2 m for estimation of mean fruit weight. These samples were intended as a randomly selected sample, but it was noted that conscious attempts to select small numbers of representative samples from a large population are unreliable. The estimated mean was within ≈13% of the true mean, measured from the harvesting of all fruit on a tree. 

**Figure 11:**
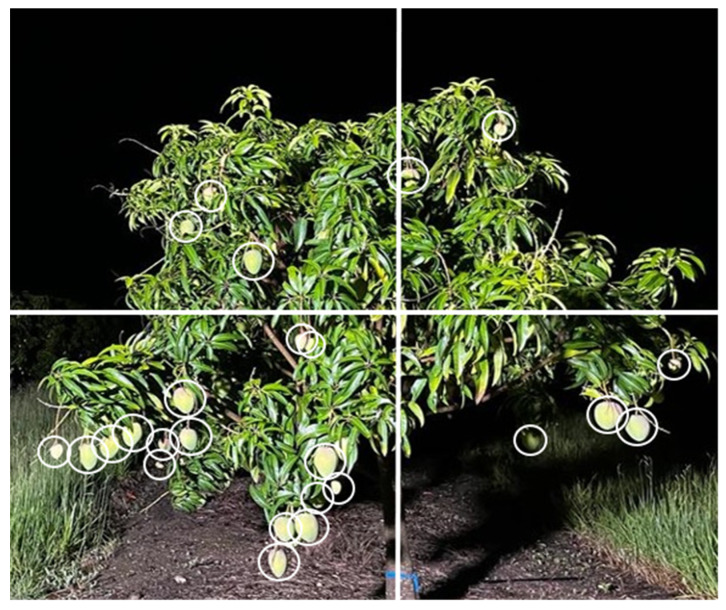
Selecting representative fruit for size estimation. Image of one face of a mango tree with all fruit labelled, divided into four quarters for sub-sampling. Alternatively, a mobile phone app is proposed for fruit detection in the image, followed by random selection of a user defined number of fruit for manual assessment.

One approach for creating smaller sampling units for selection of a random sample of fruit is to visually divide each tree face (Figure 11) into units from which a sample is then ‘randomly’ selected for sizing, with a different canopy face and unit used for each tree sampled. However, tree sections with less fruit will be over-represented in such a sample. A future aid to sampling would be a mobile phone app that enabled detection of fruit in the collected image and the random selection of a user-defined number of fruit. Another approach would be to undertake assessment of all fruit on the selected tree face. A 3D machine vision method is currently used to assess a size profile of fruit in field bins (approximately 1.2 × 1.2 m) from images collected using a mobile device (see Section 4.5). This method could be developed for assessment of the size of fruit on sampled trees.

Other considerations apply for the use of ‘drive-by’ machine vision implemented on a mobile platform. Operator effort can be reduced by driving a sample of inter-rows in the orchard, with a systematic sampling easiest to apply, e.g., driving of every third row, as recommended in a fruit count application [99]. Furthermore, not all fruit on the canopy is visible in the acquired images, and the field of vision of the camera may not be set to capture the whole canopy. Thus, the representativeness of the visible fruit as a sample of the total fruit population should be confirmed.

### 5.2. Sample Size

The sample number required to adequately represent a population is independent of the size of the population, but rather it is dependent on the variance of the population and the level of error that the operator is prepared to accept. For samples selected by simple random sampling, this number can be estimated from a rearrangement of the confidence interval calculation Walsh, McGlone, and Wohlers [93], and Piepho et al. [100]:

(1) $$n=\frac{\sigma^{2}{\left(t_{\alpha /2,n-1}\right)}^{2}}{e^{2}}={\left(\frac{\sigma t_{\alpha /2,\;n-1}}{e}\right)}^{2}$$

where σ is the sample standard deviation, t is the t-statistic, and e is the accepted error. As t requires an estimate of degrees of freedom that, in turn, requires a value for n, the Z-score can be used as an initial estimate.

For example, consider an orchard with 1000 trees in which 50 fruit are selected randomly and sized, with an estimated mean weight of 485 g and SD of 25 g per fruit. The number of fruit that should be counted to achieve a margin of error of 5 g per fruit with 95% confidence (a = 0.05) can be estimated using a Z-score lookup table (z = 1.961) as:

$$n={\left(25\times 1.961÷5\right)}^{2}=96.1$$

Using n = 96, t can then be obtained from a lookup table (using 96 – 1 = 95 degrees of freedom) as 1.985. The n requirement can then be re-estimated as 98.5. As 98.5 is larger than 96.1, the process should be repeated, with the resulting solution being 98.4. This can be accepted as the recommended minimum number of fruit required to estimate population parameters. 

Note that a first sampling is required to achieve an estimate of the population SD for the above calculations. In practice, this estimate may be based on prior experience, e.g., from previous seasons or orchards. Alternatively, smart devices can enable an on-the-go calculation of the required n using the above equations, as measurements are made in the field. 

If the calculated sample size is within 5% of the population size, a finite population correction adjustment should be made, as:

(2) $$n\left(FPC\right)=\frac{nN}{n+\left(N-1\right)}$$

The above discussion pertains to sampling by simple random sampling. Other calculations apply for estimation of minimum sample size in context of other sampling strategies [93], e.g., systematic [95], stratified [96,101], and cluster [102]. Equation 1 can be used to estimate the minimum required sample number within each cluster or strata, but (fruit size) population parameters estimated using samples from these clusters or strata must be weighted for the number of fruit in each cluster or strata. A useful summary is provided by Thompson [103], and online calculators specific to sampling strategy can be found, e.g., https://stattrek.com/survey-sampling/sample-size-calculator.aspx?tutorial=samp (Accessed on: 1 April 2023).

In summary, for manual estimation methods, either traditional or machine vision based, the use of a SUR sampling strategy and a sample number calculated from preliminary estimates of population SD is recommended. Drive-by machine vision is convenient to undertake on a row basis, i.e., image the length of a full row, with a systematic sampling of rows recommended, e.g., every fifth row, after some assessment of variation across the orchard. However, the drive-by imaging estimate is based on non-occluded fruit only, so a check is required on the representativeness of this sample.

## 6. Forecast of Harvest Weight

Fruit load forecasts are required as early as possible before harvest to inform management decisions. In general, fruit numbers are typically relatively static in the month before harvest, but fruit size typically continues to increase. A growth model is, therefore, required to allow forecasts of fruit weight at harvest from measurements made some time earlier.

Two distinct approaches can be taken in order to model fruit growth, viz. mechanistic and empirical. A mechanistic model is built on an understanding of tree physiology, e.g., a mechanistic model of mango fruit growth has been developed based on input of environmental data and modelling of processes, such as source–sink relationship, mobilization of reserves, and respiration [104] and water relations [105]. An empirical model is a function fitted to the available data. Given their relative simplicity to develop and implement, focus is given to empirical modelling here. 

**Figure 12:**
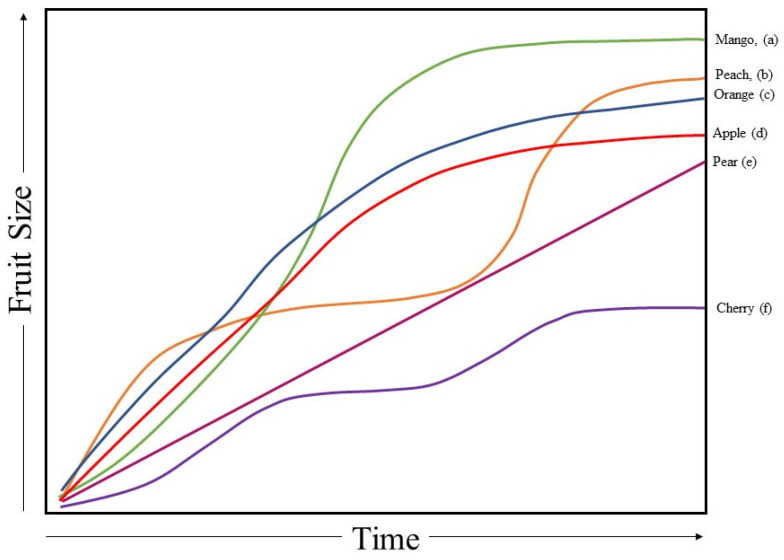
Typical growth curves for lineal dimensions (diameter or length) from fruit set to harvest for different commodities, compiled from (a) [108], (b) [109], (c) [110], (d) [111], (e) [112], and (f) [113]. Colored lines are aligned to fruit type.

The trajectory of fruit growth varies between species (Figure 12). A number of mathematical models have been used in the description of these trajectories (Table 5). The overall time course of fresh weight gain during fruit development is typically described by a single or a double logistic sigmoidal function. A sigmoidal function is characterized by slow initial growth followed by acceleration in growth, and then a de-acceleration towards maturity. The early period can be characterized as exponential, where the size of the fruit changes at an increasing rate over time, followed by a convex decrease, with the last period approximated by a linear rate of increase, a plateau or even a decrease [106]. As an alternative to a logistic sigmoidal curve, which is symmetrical, the asymmetrical Gompertz or Richards functions are sometimes employed [107]. Two such functions can be summed to model a double curve, such as displayed by peach in Figure 12 (see also the form of the equation for peach in Table 6).

Some mechanistic explanation of the shape of a sigmoidal curve is offered by the description of fruit growth as occurring in three growth stages: Stage I (cell division), Stage II (cell expansion) and Stage III (maturation and reserve accumulation) [114]. The weight or volume growth curve of a fruit is complex in that the fruit contains several tissues, each developing separately. There can be a change in tissue porosity (% airspace) and in storage product and structural carbohydrate (e.g., endocarp thickening) content which dry matter content and volume to weight ratio. 

**Table 6:** Example growth models for fruit size (weight or lineal dimension).

**Commodity-Cultivar**	**Model**	**R^2^**	**MODEL TYPE**	**Ref.**
Apple *-Gala*	$W_{h}=-169.09+\left(8.19\times W_{60\;DAB}\right)$	0.79	Linear	[5]
* -Gala*	$FD=3.5+88.23\times {\left(1-e^{0.021x}\right)}^{2.286}$	0.99	Gompertz	[115]
* -Jerseymac*	$FD=-6.68257+1.12916\times t-0.00374\times t^{2}$	0.99	Sigmoidal	[111]
* -Galaxy Gala*	$FD=-11.88672+0.96623\times t-0.00254\times t^{2}$	0.99	Sigmoidal	
* -Braeburn*	$FD=-2.0434+0.69868\times t-0.0016\times t^{2}$	0.99	Sigmoidal	
Kiwifruit -*Hort16A*	$W_{h}=a[m_{0}\exp\left(m_{1}t-m_{2}t^{2}\right)+(1/(1+m_{3}/t^{k}))]$	0.99	-	[116]
Blackberry -*Guarani*	$FD=\frac{12.74}{\left(1+e^{3.58-0.11\times t}\right)}+\frac{16.52-12.74}{\left(1+e^{32.94-0.43\times t}\right)+\varepsilon_{i}}$	0.98	Double logistic	[117]
Papaya -*Siluet*	$FD=70.27\times e^{-e^{0.23-0.0038\times x}}$	0.99	Gompertz	[118]
* -Red Lady*	$FD=84.19\times e^{-e^{0.07-0.0037\times x}}$	0.99	Gompertz	
Peach -*Aurora-1*	$FD=26.22+e^{0.1868\times \left(28.35-t\right)}+\left(48.12-26.22\right)e^{-e^{0.1367\times \left(80.9-t\right)}}+\varepsilon_{i}$	-	Logistic + Gompertz	[109]
Strawberry -*Albion and Camarosa*	$W_{h}=458.72\times e^{-e^{\left(0.001\times \left(2782.22-x\right)\right)}+}\varepsilon_{i}$	0.99	Gompertz	[106]

Note: $W_{h}$ is weight of fruit at harvest (g), $W_{\;60\;DAB}$ is weight of fruit (g) 60 days after bloom, FD is fruit diameter (mm), x is Growing Degree Days after flowering, t is days after full bloom (DAFB) [107] or days after mid bloom (DAMB) [113] or days after anthesis [119] or days after flowering [105], a, $m_{0}$, $m_{1}$, $m_{2}$, $m_{3}$, and k are parameters established by non-linear regression and $\varepsilon_{i}$ represents random error.

A logistic sigmoidal curve with a zero asymptote has three parameters, I, the age at the inflection point, L∞, the upper asymptote (final size after infinite growing time), and t, time. Similarly, the Gompertz function uses three parameters, L∞; the difference between initial and final diameter over time, and the specific growth rate, k. 

The Richard’s function employs four parameters, L∞; k; γ, the point of inflection on the x axis and σ, a parameter that in part determines the point of inflection on the y axis.

Examples of reports presenting fruit growth models are presented in Table 6. Such functions can be used to describe an existing growth dataset. However, their use to predict growth in another season or location requires consideration of the impact of environmental variations on input parameters, such as L∞. This can be addressed by incorporating the environmental variable; e.g., Hsieh et al. [120] recommended development of sigmoidal models of growth against thermal rather than calendar time, with the use of season-specific models despite incorporation of temperature into the model. 

In another approach, a cubic smoothing splines function was used for the prediction of the harvest weight of navel oranges using a model based on data from three growing seasons [121]. Predictions of average fruit size at harvest made at early Stage II (cell enlargement) of fruit development, 5–6 months prior to harvest, achieved an R^2^ value of 0.83, 0.85, and 0.81 on actual harvest weight in the three respective seasons. A three-class fruit size distribution was reported to be not significantly different for the Stage II prediction and harvest populations, but an R^2^ value on predicted and actual size was not presented. As the study involved healthy, well-irrigated trees growing in one location, with no differences in fruit growth rate for the same measurement dates across the seasons, further validation is recommended. 

Working with five seasons data across two sites, Minchin, Richardson, Patterson, and Martin [116] described an increase in kiwifruit fresh weight using an equation of the form presented in Table 6, with fruit weight predicted for a given time after mid-bloom in calendar days (DAMB). Prediction of final fruit weight (at 200 DAMB) from a measurement after 80 DAMB was reported to be independent of season and site, with predictions within 6% of final fruit weight. It was postulated that the potential maximum kiwifruit weight is determined by 80 DAMB, irrespective of subsequent climate. Initial fruit size was also found to be a clear determinant of fruit size at harvest in four cultivars of papaya by Salinas, Hueso, and Cuevas [118]. Thus, the trajectory of late fruit weight and volume may be altered by environmental factors in the short term but no longer than that. For example, a size increase, and dry matter content plateau, in mango fruit associated with a rainfall event was of short-term impact [99].

Other approaches to predictive modelling of fruit growth deserve further attention, including the concept of fruit biological age [122] and the use of quantile functions in the prediction of future fruit population distributions and of the calendar date (or degree days) at which a specified proportion of the fruit population meets a specified target [123].

## 7. Conclusions

As fruit value chains increase in length and scale, the need for the forecast of fruit size at harvest increases. This requires the availability of both robust allometric relationships between fruit weight, volume, and lineal dimensions, and of robust growth models. These relationships and models are specific to commodities and cultivars. Field measurements must also conform to statistically valid sampling designs, which must also be designed to be as efficient as possible. Substantive work has been performed in all areas for certain commodities, but work remains to extend to develop these aids for all commodities. Effort is also required in the development of protocols that can be practically implemented by producers seeking a forward estimate of fruit size distribution at harvest.

At the core of a sizing forecast is the need to measure the size of fruit on-tree. Fruit lineal dimensions can be measured non-destructively using either traditional caliper technology or machine vision. The motivation for the use of machine vision in fruit sizing is to attain decreased sampling effort.

Progress in the development of machine vision and its application to fruit sizing has been notable in the 14 years since the Moreda, Ortiz-Cañavate, García-Ramos, and Ruiz-Altisent [2] review. Several ‘generations’ of approaches to the issue of image scale have occurred, moving from the use of a fixed camera to object distance to use of a scale placed in the object plane and more recently to concurrent measurement of the camera to the object distance through the use of RGB-D cameras. Several ‘generations’ have also passed in terms of object detection and segmentation, from simple color thresholding to deep learning detectors and segmentors. An area deserving of continued attention is that of the discrimination of partly occluded from non-occluded fruit. Another area deserving of continued attention involves the use of 3D reconstruction in fruit sizing. The claims by several groups for lineal dimension RMSE of 1–3 mm using 3D reconstructions are impressive but require further validation, and the extension from near spherical to other shaped fruit. A mobile app could be developed that provided guidance in selection (using either a random or a systematic sampling strategy) and location (using geolocation capability) of trees for sampling, and imaging of those tree canopies for a cloud-based 3D reconstruction followed by fruit sizing. 

Hardware is another area that is rapidly evolving, in terms of processing capacity and RGB-D and LiDAR technologies. The advent of low-cost RGB-D cameras, including in mobile phones, has fueled a surge in fruit-sizing applications but these units are not IP67-rated. Future developments in imaging hardware will drive the direction of developments in the fruit-sizing application area. 

Machine vision-based systems for fruit sizing are beginning to see commercial deployment. These systems are developed using hand-held smart devices and as farm vehicles or UAV-mounted hardware. Image processing can now either occur through edge computing, given improvements in computing hardware, or in the cloud, given continuing improvements in communication networks in fruit producing areas, including both improvements in 4G/5G coverage and meshed Wi-Fi systems. These systems are nascent in horticultural use, with traditional caliper technology yet dominant, but progress is rapid.

## Figures and Tables

**Table 1:** Allometric relationships between fruit weight (W, in g), volume (V, in cm^3^), and lineal dimensions (in cm) for different commodities, where FD is fruit diameter, L is fruit length, $W_{i}$ is fruit width, T is fruit thickness. R^2^ is the calibration statistics. RMSE refers to the root mean square error (g) along with the population mean weight (g).

Commodity	Cultivar	Model of Weight to Lineal Dimensions	R^2^	RMSE/Mean	Reference
Apple	Gala	$W=17.03-\left(1.48\times FD\right)+\left(0.046\times FD^{2}\right)$	0.97	-	[5]
	Fuji	$W=51.63-\left(3.691\times FD\right)+\left(0.073\times FD^{2}\right)$	0.89	-	
	Honeycrisp	$W=36.65-\left(3.311\times FD\right)+\left(0.068\times FD^{2}\right)$	0.95	-	
Avocado	Ettinger, Fuerte, Hass, Nabal, Reed	W=−313.622+2.231×L+4.572×FD	0.83	11.7/162–327	[6]
Blueberry	Bluegold	$W=12.84\times FD^{0.356}$	-	-	[7]
	Legacy	$W=12.87\times FD^{0.359}$	-	-	
Kiwifruit	Hayward	$W=0.454\times {\left(L\times FD_{1}\times FD_{2}\right)}^{1.05}$	0.99	-/90–100	[8]
	Hayward	$W=0.467\times {\left(L\times FD_{1}\times FD_{2}\right)}^{1.05}$	0.93	-	[9]
	Zesy002	$W=0.378\times {\left(L\times FD_{1}\times FD_{2}\right)}^{1.09}$	0.97	-	
Mango	Chok Anan	W=0.539×L×Wi×T	0.97	12.2/333	[10]
	Nam Dokmai	W=0.45×L×Wi×T	0.99	6.5/300	[11]
	Nam Dokmai	ANN model using L, Wi, T and area	0.99	6.6–11.8/342 g	[12]
	Honey Gold	$W=0.42\times L\times Wi^{2}$	0.96	45/420	[13]
	Honey Gold, Calypso and Keitt	W=0.5472×L×Wi×T	0.99	29.9/353–572	[14]
Nectarine	September Bright	$W=1.8\times FD^{2.709}$	0.99	2.2/-	[15]
Orange	10 cultivars	$W=0.07\times Wi^{2}-2.95\times Wi+39.15$	0.97	-	[16]
Tangerine	3 cultivars	$W=0.07\times Wi^{2}-3.78\times Wi+73.80$	0.83	-	[17]
**Commodity**	**Cultivar**	**Model of weight to volume**	**R^2^**	**RMSE/Mean (g)**	**Reference**
Citrus	Bergamot	W=0.52×V+44.72	0.99	-/292 g	[18]
Mango	Zebdia	W=1.0216×V+6.5484	0.97	9.1/379	[19]
Pomegranate	-	W=0.96×V+4.25	0.99	-/291	[20]
Tangerine	3 cultivars	W=0.99×V−5.52	0.96	-	[17]

**Table 2:** Performance metrics (see footnote for terms and Appendix A for equations) and method for reports on machine vision-based fruit sizing, categorized by fruit type.

Fruit	Studies	Metrics	Value	Device/Method	2D/3D
apple	[43]	RMSE	1.79 mm (diameter)	Low-cost color camera used, packhouse application for grading and sorting, vision processing algorithm for real time estimation of orientation, shape, and size of fruit.	2D
	[44]	RMSE	1.79 mm (diameter)	Packhouse application using color camera and image processing with thresholding based on color/intensity.	2D
	[25]	MAPE MAPE	15.2% (using pixel size) 30.9% (using 3D cord.)	PMD CamCube 3.0 ((PMD Technologies, Siegen, Germany) ToF camera and a color camera used, in orchard sizing, Hough circular detection, and Otsu thresholding used for segmentation.	2D, 3D
	[45]	RMSE RMSE	<1.0 mm (diameter), <6 cm3 (volume)	Reconstruction of fruit shape using 3D point clouds from RGB-D camera.	3D
	[42]	RMSE	5.1 mm (at >40% visibility)	Photogrammetry Structure from Motion (SfM) method used, and M-Estimator sample consensus (MSAC) method proved lowest error among four different methods evaluated.	3D
	[27]	RMSE RMSE	10.41 mm (oblique image) 11.01 mm (panoramic image)	RGB image, YOLO based detection model, Otsu thresholding, and reference object on scene.	2D
	[46]	MAE MAPE	1.04 mm 9.35%	RealSense L515 (Intel, Santa Clara, USA) ToF camera used in orchard, YOLACT segmentation model, DBSCAN tool for point cloud clustering, and ellipse fitting for sizing.	3D
mango	[10]	MPE	29% (mass overestimation)	Fruit circumference manually measured from digital images.	2D
	[13]	RMSE RMSE	4.9 mm (length) 4.3 mm (width)	Kinect v2 RGB-D (Microsoft, Washington, USA) ToF camera used, in orchard imaging, HOG cascade detector for fruit detection, Otsu thresholding used with stalk removal filtering.	2D
	[23]	RMSE RMSE	5.3 mm (length) 3.7 mm (width)	Smartphone application used with image taken by phone camera and Otsu thresholding used for segmentation.	2D
	[12]	RMSE RMSE	10.38 g (mass from pixel area from 1 camera) 8.17g (mass from L, W and T using top and side camera)	Color webcams used for imaging from top and side in lab setup, color-based segmentation for binary mask, features such as enclosing rectangle, mask area and fit ellipse properties used for sizing assessment.	2D
	[47]	R^2^	0.997 (size estimation)	A general CCTV color IP camera used for continuous image acquisition, color-based image thresholding followed by morphological operation and randomized Hough transform to create mask and sizing from masked segment.	2D
	[48]	R^2^ R^2^	0.991, 0.96, 0.954 (L, W, T for Chausa cultivar) 0.981, 0.967, 0.95 (L, W, T for Dashehari cultivar)	Monochrome images used and HIS color image used for segmentation and dimension from contour used for size estimation.	2D
	[40]	RMSE R^2^	4.7 mm (length) 0.9 (length)	Azure Kinect (Microsoft, Washington, USA) RGB-D ToF camera used, in orchard imaging, Mask R-CNN for instance segmentation, occlusion avoidance with conditions.	2D
citrus	[49]	RMSE R^2^	10 mm 0.8085	Color based segmentation of image, packhouse application, used in a portable fruit grading system.	2D
	[50]	Accuracy	100% (grading accuracy based on size using radius signature)	Color images on plain background taken in the lab are segmented using contour detection and sizing methods assessed based on radius signature, area, perimeter, and using laser rangefinder’s LiDAR data. Overall grading accuracy based on estimated size is reported.	2D, 3D
	[23]	RMSE RMSE	3.8 mm (for mandarin) 2.4 mm (for navel orange)	Smartphone application, image taken by phone color camera, Otsu thresholding used for segmentation, reference scale used in image for conversion of size in pixel to metric size.	2D
	[51]	MAD	4 mm	Kinect v2 RGB-D camera, filtering with probabilistic density function, point cloud clustering, SVM classifier used.	3D
	[37]	N/A	sizing distribution against manually measured data reported	RGB image from UAV analyzed with reference scale in the image background, Faster R-CNN for detection, color-based thresholding used to create segmentation mask and lineal dimensions taken from mask.	2D
tomato	[52]	Efficiency	90% (size)	Color based thresholding applied on color images taken in the lab at known distance from camera, dimension of the segmented fruit pixels converted to mm size.	2D
	[53]	MAPE	7.09% (for mass)	RGB images taken in controlled light environment, known distance, Mask R-CNN segmentation, size from segmented mask.	2D
	[54]	R^2^ R^2^	0.9041 (factor for width) 0.9344 (factor for height)	ZED mini camera used in orchard for imaging, with Mask R-CNN segmentation used. Conversion factors for width and height estimation from images are reported.	2D
	[55]	MAPE MAPE	7.53% (for diameter) 11.63% (for length)	RealSense D435i and D455 RGB-D (Intel, Santa Clara, USA) cameras used for imaging, Keypoint R-CNN model used for key point detection, dimensions estimated from key point’s 3D positions.	3D
pear	[41]	RMSE RMSE	1.17 mm (vertical diameter) 1.03 mm (horizontal diameter)	Kinect v2 RGB-D camera used, 360-degree surface point cloud obtained, 3D fruit constructed and stalk removal algorithm applied, fruit dimensions taken from a 3D bounding box enclosing constructed 3D fruit from point clouds.	3D
	[56]	N/A	No sizing accuracy reported	U-Net based semantic segmentation model applied to RGB image at various scaled image and growth rate monitored using segmented mask pixels on time series image data.	2D
pepper	[57]	Accuracy	56% (volume)	RealSense L515 RGB-D camera, instance segmentation with YOLACT model used and segmentation ROIs compared with ground truth ROIs, two methods assessed where ‘unobserved voxel utility’ method provided highest accuracy.	3D
	[55]	MAPE MAPE	10.8% (diameter) 14.5% (length)	RealSense D415 (Intel, Santa Clara, USA) RGB-D camera, Keypoint R-CNN model used for key point detection, 3D coordinates of key points used for sizing.	3D
cucumber	[58]	ME R^2^	3.20% (volume) 0.9805 (volume)	RGB image acquired indoors at known camera distance, Otsu thresholding for segmentation, masks enclosing bounding box dimensions with slicing approach used for sizing.	2D
	[55]	MAPE MAPE	18.44% (diameter) 14.17% (length)	RealSense D415 RGB-D camera, Key point R-CNN model used for key point detection, 3D coordinates of key points used for sizing.	3D
olive	[59]	RMSE RMSE	0.178 g (using Arbequina data) 0.2439 g (using Picual data)	RGB image acquired indoors at known camera distance, converted to HSV color space, noise reduced, contrast increased, Otsu’s thresholding for segmentation, structuring element filter for noise removal, major and minor axis length use for size estimation.	2D
	[60]	Relative error	<1.16% (mass estimation)	RGB image acquired indoors at known camera distance, of clustered olives, segmented with watershed transformation and morphological transformation.	2D
passion fruit	[61]	SE	31.58 cm3 (volume on test images)	RGB image acquired indoors at known camera distance, segmentation and contour detection and enclosing bounding box on segmentation mask used for sizing in terms of mass and volume, Trained Artificial Neural Network (ANN) used as an estimation tool.	2D
grape	[62]	Accuracy	92.1% for grading by size	Packhouse application uses RGB and IR images taken at known camera distance, color, and intensity-based thresholding for edge detection.	2D
	[63]	MAE	2.9 cm (cluster length) 3.6 cm (cluster width)	RealSense D435 RGB-D camera, point cloud clustering to extract grape cluster, bounding box, ellipsoid and cylinder fitting on cluster for sizing.	3D
melon	[64]	MAE	<50 g (weight)	RGB image taken at known camera distance, image segmented by the contour of larger area, weight predicted from area of segmented pixels.	2D
onion	[65]	RMSE RMSE	3.4 mm (diameter) 18.5 cm3 (volume)	Kinect RGB-D camera used for imaging, Otsu thresholding for segmentation of onion region, point cloud analysis to fit ellipsoid model, ellipse and ellipsoid major axis length used for sizing.	3D
pomegranate	[66]	R^2^	0.97 (volume)	X-ray computed tomography imaging used for segmentation of pomegranate fruit, 3D image reconstructed for volume estimation.	3D
banana	[38]	RMSE	1.68% (volume)	CycleGAN model trained on banana image sets to generate enhanced banana fruit 2D and reconstructed 3D models for fruit volume estimation.	3D
pumpkin	[67]	R	0.844 (for volume at 99% confidence level)	RGB images from UAV used, binary classification with morphological transformation used, ellipsoid model applied to estimate volume of the fruit.	2D
eggplant	[55]	MAPE MAPE	13.5% (diameter) 7.43% (length)	RealSense D415 RGB-D camera used, Keypoint R-CNN model used for key point detection, 3D coordinates of key points used for sizing.	3D
sweet potato	[39]	Accuracy	96% (volume with R^2^ = 0.98) 95% (weight with R^2^ = 0.96)	RGB image acquired indoors, color-based thresholding applied, enclosed bounding box on segmentation mask applied and chopped pyramid method used for estimation of size, volume, and mass.	2D
carrot	[58]	ME R^2^	3.42% (volume) 0.98 (volume)	RGB image acquired indoors, Otsu thresholding for segmentation, masks enclosing bounding box dimensions with slicing approach used for sizing.	2D

Abbreviations: RMSE—root mean square error; MAE—mean absolute error; MAPE—mean absolute percentage error; MPE—mean percentage error; SD—standard deviation; SE—standard error MAD- median absolute deviation, R^2^—coefficient of determination, R -correlation coefficient, ME-mean error, N/A—Not available.

**Table 3:** Categorization of published papers reporting fruit sizing by segmentation method.

Segmentation Method	Publication
2D	
Color/Intensity based method (OpenCV)	[39,42,48,49,50,63,67,85]
Otsu’s method (OpenCV)	[13,23,25,27,59,65]
Semantic segmentation (CNN based)	[56]
Instance segmentation (CNN based)	[46,53,54,57]
3D	
Point cloud clustering	[41,45,46,55,63]
3D shape fitting	[28,41,42]

**Table 4:** Commercially available machine vision systems for fruit sizing applications. All web links were accessed on 8 March 2023. N/A is not available.

Company (Product)	Use Case	Hardware	Depth	Processing
Hectre (Spectre) https://www.hectre.com/ (accessed on 8 March 2023)	harvest bins—manual harvest bins on truck	phone or tablet fixed camera on gantry		cloud
Croptracker (Harvest Quality Vision) https://www.croptracker.com/ (accessed on 8 March 2023)	harvest bin—manual	tablet	RGB-D	cloud, 3D reconstruction from multiple images
Pixofarm https://www.pixofarm.com/ (accessed on 8 March 2023)	fruit on tree—manual	phone or tablet	scale	N/A
FruitScout https://fruitscout.ai/ (accessed on 8 March 2023)	fruit and trunk size	phone or tablet	scale	cloud
Aerobotics https://www.aerobotics.com/ (accessed on 8 March 2023)	fruit (citrus) on tree—manual, with sampling location guided by UAV image	iPhone	RGB-D	cloud
Tevel https://www.tevel-tech.com/ (accessed on 8 March 2023)	fruit sized at moment of harvest	UAV mounted	RGB-D	N/A
Green Atlas https://greenatlas.com/ (accessed on 8 March 2023)	whole farm assessment	vehicle mounted RGB camera and LiDAR	LiDAR	cloud

**Table 5:** Mathematical models used for predicting fruit weight (W, in g).

Model	Equation	Terms
Gompertz	$W_{t}=W_{\infty }e^{e^{-k\left(t-I\right)}}$	$W_{\infty }$ is upper asymptote, k is growth rate, I is age at inflection point
Linear	$W_{t2}=a+bW_{t1}$	a is intercept, b is slope
Logistic	$W_{t}=W_{\infty }\;/\left(1+e^{-k\left(t-I\right)}\right)$	
Richards	$W_{t}=W_{\infty }{\left[1+\left(\delta -1\right)e^{-k_{i}\left(t-\gamma \right)}\right]}^{1/\left(1-\delta \right)}$	γ is age at inflection point, δ in part determines the point of inflection on y axis

## Data Availability

Not applicable.

## Appendix A

Equations used in the text:

Root Mean Square Error (RMSE): $\sqrt{\frac{\sum_{i=1}^{n}{\left(y-y_{i}\right)}^{2}}{n}}$, where y is actual value, $y_{i}$ is predicted value and n is number of samples.

Mean Absolute Error (MAE): $\frac{\sum_{i=1}^{n}\left|y-y_{i}\right|}{n}$, where y is actual value, $y_{i}$ is predicted value and n is number of samples.

Mean Absolute Percentage Error (MAPE): $\frac{100}{n}\overset{n}{\underset{i=1}{\sum }}|\frac{y-y_{i}}{y}|$, where y is actual value, $y_{i}$ is predicted value and n is number of samples.

Mean Percentage Error (MPE): $\frac{100}{n}\overset{n}{\underset{i=1}{\sum }}\frac{y-y_{i}}{\bar{y}}$, where y is actual value, $y_{i}$ is predicted value, $\bar{y}$ is mean of sample and n is number of samples.

Standard Deviation (SD): $\sqrt{\frac{\sum {\left(y_{i}-\bar{y}\right)}^{2}}{n}}$, where $y_{i}$ is predicted value, $\bar{y}$ is sample mean and n is number of samples.

Standard Error (SE): $\frac{\sigma }{\sqrt{n}}$, where σ is standard deviation and n is number of samples.

Median Absolute Deviation (MAD): $Median\;\left(\left|y_{i}-\bar{y}\right|\right)$, where $y_{i}$ is predicted value, $\bar{y}$ is sample mean.

Coefficient of Determination (R^2^): $1-\frac{Sum\;of\;squares\;of\;residuals\;\left(RSS\right)}{Total\;sum\;of\;squares\;\left(TSS\right)}=1-\frac{\sum_{i=1}^{n}{\left(y-y_{i}\right)}^{2}}{\sum_{i=1}^{n}{\left(y-\bar{y}\right)}^{2}}$, where $\bar{y}$ is sample mean, *n* is number of samples, $y_{i}$ is instance of sample *y*.

Correlation Coefficient (R): $\frac{\sum_{i=1}^{n}\left(x_{i}-\bar{x}\right)\left(y_{i}-\bar{y}\right)}{\sqrt{\sum_{i=1}^{n}{\left(x_{i}-\bar{x}\right)}^{2}\sum_{i=1}^{n}{\left(y_{i}-\bar{y}\right)}^{2}}}$, where $\bar{x}$ and $\bar{y}$ are sample means, n is number of samples, $x_{i}$ and $y_{i}$ are instances of samples *x* and *y*.
